# An exploratory study investigating biomarkers associated with autoimmune pulmonary alveolar proteinosis (aPAP)

**DOI:** 10.1038/s41598-022-11446-8

**Published:** 2022-05-24

**Authors:** Ilaria Campo, Federica Meloni, Martina Gahlemann, Wiebke Sauter, Carina Ittrich, Corinna Schoelch, Bruce C. Trapnell, Abhya Gupta

**Affiliations:** 1 UOS trasnplant center and Pneumology Unit, Fondazione IRCCS Policlinico San Matteo, Pavia, Italy; 2 Department of Internal Medicine, University of Pavia, Pavia, Italy; 3 Boehringer Ingelheim (Schweiz) GmbH, Basel, Switzerland; 4 Boehringer Ingelheim Pharma GmbH & Co. KG, Biberach an der Riss, Germany; 5 Translational Pulmonary Science Center, Cincinnati Children’s Hospital, Cincinnati, OH USA; 6 Boehringer Ingelheim International GmbH, Biberach an der Riss, Germany

**Keywords:** Biomarkers, Respiratory tract diseases

## Abstract

Autoimmune pulmonary alveolar proteinosis (aPAP) is a rare lung disorder involving production of autoantibodies against endogenous granulocyte–macrophage colony-stimulating factor (GM-CSF). This study aimed to identify biomarkers that could be used to monitor for aPAP, particularly in patients treated with anti-GM-CSF antibodies. This was an exploratory, prospective, observational, single-center study. Pre-specified biomarkers were evaluated between baseline and Day 120 in serum/plasma, whole blood, sputum and exhaled breath condensate from patients with aPAP, healthy volunteers, and patients with chronic obstructive pulmonary disease (COPD) and asthma (not treated with anti-GM-CSF and with no evidence of aPAP). Pulmonary function tests were also performed. Overall, 144 individuals were enrolled (aPAP: n = 34, healthy volunteers: n = 24, COPD: n = 40 and asthma: n = 46). Plasma GM-CSF levels were lower, and Krebs von den Lungen 6 and GM-CSF autoantibody ranges were higher, in patients with aPAP compared with other populations. Surfactant proteins-A and -D, lactate dehydrogenase and carcinoembryonic antigen ranges partially or completely overlapped across populations. Most plasma biomarkers showed high sensitivity and specificity for detection of aPAP; GM-CSF and GM-CSF autoantibody concentrations demonstrated equivalent sensitivity for differentiating aPAP. In addition to characteristic GM-CSF autoantibodies, assessment of plasma GM-CSF may identify individuals at risk of developing aPAP.

Trial registration: EudraCT, 2012-003475-19. Registered 23 July 2012—https://eudract.ema.europa.eu/.

## Introduction

Autoimmune pulmonary alveolar proteinosis (aPAP) is a rare lung disorder associated with production of antibodies against endogenous granulocyte–macrophage colony-stimulating factor (GM-CSF) autoantibodies. It is the most common pulmonary alveolar proteinosis-causing disease, accounting for up to 90% of cases^1–4^. GM-CSF has several crucial roles in health, including alveolar macrophage differentiation, alveolar stability, lung function, host defense and surfactant homeostasis. By neutralizing GM-CSF, GM-CSF autoantibodies in aPAP reduce pulmonary surfactant catabolism and lead to pathologic surfactant accumulation, which may contribute to respiratory failure and pulmonary fibrosis^1–3^. In addition to regulating the surfactant clearance capacity of alveolar macrophages, GM-CSF also has a role in modulating inflammatory disorders such as asthma, chronic obstructive pulmonary disease (COPD) and autoimmune diseases^5,6^.

Anti-GM-CSF antibody therapies are currently in clinical development to treat inflammatory diseases such as rheumatoid arthritis^7^. However, given the link between increased levels of endogenous anti-GM-CSF antibodies and aPAP^8^, patients receiving anti-GM-CSF therapy are theoretically at risk of developing iatrogenic aPAP.

Several clinical chemistry markers can support aPAP diagnosis^4,9–15^, but none are used on their own for this purpose and several are abnormal in other lung diseases^6,10,16–18^. Identifying a biomarker (or biomarker combination) indicative of aPAP would facilitate early identification of disease by triggering appropriate medical work-up to confirm or reject diagnosis, and could help differentiate aPAP from other respiratory diseases.

This exploratory study was undertaken to determine levels and ranges of biomarkers potentially associated with aPAP, which could be used alone or in combination to monitor for pulmonary alveolar proteinosis onset in healthy human subjects or in patients with underlying respiratory diseases receiving anti-GM-CSF antibodies therapeutically. Findings will also extend understanding of the potential risk of iatrogenic pulmonary alveolar proteinosis in patients receiving anti-GM-CSF antibodies therapeutically and support clinical development of these treatments.

## Results

### Study participants

**Figure 1:**
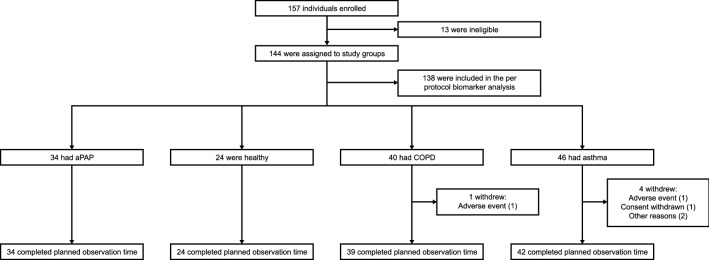
Study groups and disposition. aPAP, autoimmune pulmonary alveolar proteinosis; COPD, chronic obstructive pulmonary disease.

Altogether, 144 individuals (of 157 enrolled) met inclusion/exclusion criteria and were entered into the study (Fig. 1). Five individuals (3.5%) withdrew prematurely, two due to adverse events. Six individuals were excluded from the per-protocol biomarker analyses due to violation of study criteria, leaving 138 participants: 33 with aPAP, 24 healthy volunteers, 36 patients with COPD and 45 patients with asthma.

Baseline demographic characteristics are shown in Table 1. All but one participant was Caucasian. Apart from patients with asthma (45.7%), men made up the majority of each group (64.7–75.0%). The proportion of current or ex-smokers was higher among patients with aPAP than healthy volunteers. Patients with COPD were generally older and had worse lung function than the other populations. Exclusion criteria for patients with COPD (never-smokers and patients aged < 40 years ineligible) and patients with asthma (current smokers ineligible) were reflected in study group characteristics.

**Table 1:** Demographic, disease severity and pulmonary function characteristics at baseline.

	aPAP (*n* = 34)	Healthy (*n* = 24)	COPD (*n* = 40)	Asthma (*n* = 46)
Gender, *n* (%) male	22 (64.7)	16 (66.7)	30 (75.0)	21 (45.7)
Race, *n* (%)				
Caucasian	33 (97.1)	24 (100)	40 (100)	46 (100)
Asian	1 (2.9)	0	0	0
Age, mean (SD) years	48.1 (12.6)	39.0 (14.8)	62.5 (5.5)	46.2 (13.5)
BMI, mean (SD) kg/m^2^	25.7 (3.7)	23.7 (3.5)	26.7 (4.3)	25.0 (4.0)
Smoking status, *n* (%)				
Never smoked	10 (29.4)	14 (58.3)	0	33 (71.7)
Ex-smoker	15 (44.1)	5 (20.8)	24 (60.0)	13 (28.3)
Current smoker	9 (26.5)	5 (20.8)	16 (40.0)	0
Severity, *n* (%)				
Mild	15 (44.1)	–	10 (25.0)	11 (23.9)
Moderate	13 (38.2)	–	17 (42.5)	15 (32.6)
Severe	–	–	8 (20.0)	11 (23.9)
Severe exacerbation	–	–	5 (12.5)	9 (19.6)
Stable remission	6 (17.6)	–	–	–
DL_CO_% predicted, mean (SD) %	64.6 (16.9)	88.3 (14.6)	68.5 (19.8)	91.2 (15.2)
DL_CO_ corrected for Hb, mean (SD) mmol/min/kPa	3.8 (1.1)	5.7 (1.4)	3.5 (1.1)	5.3 (1.6)
FEV_1_, mean (SD) L	2.91 (0.81)	3.86 (0.86)	1.65 (0.59)	2.85 (0.85)
FEV_1_% predicted, mean (SD) %	89.8 (19.4)	106.1 (8.6)	61.1 (20.7)	90.4 (16.8)
FVC% predicted, mean (SD) %	93.4 (20.5)	110.0 (11.2)	84.2 (15.5)	105.2 (15.2)

*aPAP* autoimmune pulmonary alveolar proteinosis, *BMI* body mass index, *COPD* chronic obstructive pulmonary disease, *DL*_*CO*_ diffusing capacity of the lungs for carbon monoxide, *FEV*_*1*_ forced expiratory volume in 1 s, *FVC* forced vital capacity, *Hb* hemoglobin, *SD* standard deviation.

### Clinical outcomes

#### Symptom severity

Baseline severity classification of patients is shown in Table 1. For patients with asthma or COPD, disease severity or severe exacerbation was related to higher scores on symptom questionnaires (corresponding to uncontrolled symptoms/worse health).

#### Pulmonary function

Mean lung function parameters at baseline differed between the four study populations (Table 1). Diffusing capacity of the lungs for carbon monoxide (DL_CO_) % predicted and forced vital capacity (FVC) % predicted were higher in healthy volunteers (88.3% and 110.0%) and patients with asthma (91.2% and 105.2%) than in patients with COPD (68.5% and 84.2%) or aPAP (64.6% and 93.4%). Forced expiratory volume in 1 s (FEV_1_) was normal in healthy volunteers (3.86 L). Mean FEV_1_ values were lower but still within normal ranges in patients with asthma or aPAP (2.85 L and 2.91 L), and below normal in patients with COPD (1.65 L). Lung function was related to disease severity for patients with aPAP or COPD, but not for patients with asthma. Lung function parameters remained consistent over the 120-day observation period (Additional File 1: Table S2).

### Biomarkers as identifiers of aPAP

Inter-individual variability was generally higher in induced sputum samples than in serum/plasma/whole blood samples (Table 2).

**Table 2:** Absolute values for biomarkers at baseline (per-protocol biomarker analysis, mean (SD) [95% reference interval]*).

	aPAP (*n* = 33)	Healthy (*n* = 24)	COPD (*n* = 36)	Asthma (*n* = 45)
KL-6/plasma, U/mL	3670.4 (3282.2)	244.7 (74.8)	289.2 (144.9)	299.7 (128.1)
	[489.9–9506.3]	[88.0–406.7]	[95.0–573.2]	[114.9–572.7]
SP-A/serum, ng/mL	84.8 (54.4)	133.7 (238.2)	56.1 (39.4)	40.5 (31.8)
	[13.2–244.2]	[9.41–NA]	[19.8–179.4]	[15.8–120.3]
SP-D/plasma, ng/mL	313.9 (191.5)	99.2 (66.8)	102.4 (48.0)	73.0 (39.1)
	[62.8–882.9]	[16.0–257.1]	[27.0–226.6]	[27.5–193.6]
CEA/plasma, ng/mL	31.0 (29.8)	18.7 (26.2)	22.6 (16.1)	23.6 (17.0)
	[2.4–134.9]	[2.1–198.5]	[2.9–73.0]	[1.8–74.8]
CYFRA/plasma, ng/mL	4.7 (4.5)	1.0 (0.6)	1.2 (0.6)	0.9 (0.3)
	[nc]	[nc]	[nc]	[nc]
LDH/serum, U/L	223.2 (47.8)	153.8 (27.9)	167.3 (27.2)	168.6 (26.2)
	[142.9–345.0]	[0–NA]	[122.7–238.0]	[117.7–224.7]
GM-CSF/plasma, pg/mL	0.2 (0.2)	1.4 (1.3)	1.1 (0.6)	1.2 (1.1)
	[0.02–0.8]	[0.6–2.2]	[0.6–2.3]	[0.7–2.1]
GM-CSF autoantibodies/plasma, ngEq/mL^†^	50,538 (52,539)	62.5 (0)	74.6 (45.3)	62.5 (0)
	[2590.4–238897]	[nc]	[nc]	[nc]
CD11b stimulation index/whole blood	0.1 (0.2)	0.7 (0.5)	0.9 (0.6)	1.0 (0.5)
	[-0.2–0.5]	[0.2–2.5]	[0.2–2.7]	[0.2–2.0]
pSTAT5 stimulation index/whole blood	-0.01 (0.2)	1.1 (0.6)	1.2 (0.7)	1.2 (0.7)
	[-0.2–0.5]	[0.2–2.9]	[0.3–3.5]	[0.4–2.0]
KL-6/sputum, U/mL	1804.0 (3016.7)	183.5 (182.8)	241.3 (327.9)	118.4 (93.7)
SP-A/sputum, ng/mL	5.4 (4.5)	2.7 (2.4)	4.1 (3.7)	1.2 (0.9)
SP-D/sputum, ng/mL	40.6 (63.4)	4.5 (9.0)	10.8 (33.6)	1.8 (2.5)
CEA/sputum, ng/mL	75.1 (141.6)	58.3 (110.0)	128.0 (147.6)	53.5 (106.1)
CYFRA/sputum, ng/mL	9.4 (20.3)	9.9 (28.2)	23.3 (38.7)	6.2 (21.8)
GM-CSF/sputum, pg/mL	3.7 (5.8)	0.06 (0.06)	0.06 (0.06)	0.08 (0.09)

Numbers of patients assessed differ slightly for some biomarkers.

*Calculation based on the robust method in the Clinical and Laboratory Standards Institute guideline^36^.

^†^Concentrations of GM-CSF autoantibodies were BLQ for all healthy volunteers, all patients with asthma and most with COPD. All these concentrations were therefore set to 0.5 LLQ, yielding mean plasma concentrations of 62.5 ngeq/mL for healthy volunteers and patients with asthma.

*aPAP* autoimmune pulmonary alveolar proteinosis, *BLQ* below the limit of quantification, *CD11b* cluster of differentiation molecule 11b, *CEA* carcinoembryonic antigen, *CI* confidence interval, *COPD* chronic obstructive pulmonary disease, *CYFRA* cytokeratin-fragment, *GM-CSF* granulocyte–macrophage colony-stimulating factor, *KL-6* Krebs von den Lungen 6, *LDH* lactate dehydrogenase, *LLQ* lower limit of quantification, *NA* calculation of upper limit not possible; *nc*, not calculated, as > 20% of data points were below or above limits of quantification, *pSTAT5* phosphorylated signal transducer and activator of transcription 5, *SD* standard deviation, *SP-A* surfactant protein-A, *SP-D* surfactant protein-D.

#### Serum/plasma/whole blood

Mean plasma GM-CSF autoantibody concentrations were high in patients with aPAP (50,538 ngeq/mL), but below limits of quantification for all healthy volunteers, all patients with asthma and most with COPD (Table 2). Plasma Krebs von den Lungen 6 (KL-6) ranges in patients with aPAP were mostly above those in healthy volunteers and in patients with COPD or asthma, whereas ranges of plasma GM-CSF and of the stimulation index based on whole blood cluster of differentiation molecule 11b (CD11b) and phosphorylated signal transducer and activator of transcription 5 (pSTAT5) expression in patients with aPAP were mostly below those in healthy volunteers and in patients with COPD and asthma (Table 2). Cut-off concentrations of these biomarkers distinguished aPAP from COPD and asthma with high sensitivity and specificity (Table 3). In contrast, surfactant protein-A (SP-A), surfactant protein-D (SP-D), lactate dehydrogenase (LDH), carcinoembryonic antigen (CEA) and cytokeratin fragment (CYFRA) ranges in patients with aPAP partially or completely overlapped those in healthy volunteers and in patients with COPD or asthma (Table 2). The observed correlations of biomarker values in blood and lung function parameters at baseline for patients with aPAP are summarized in Additional File 1: Table S3.

**Table 3:** Diagnostic and predictive power for aPAP of blood sample biomarkers (specificity ≥ 80%) [per-protocol biomarker analysis].

	aPAP vs healthy	aPAP vs COPD	aPAP vs asthma
**KL-6/plasma, U/mL**			
Cut-off value	324.30	393.60	393.00
Sensitivity (95% CI)	0.97 (0.85–0.99)	0.97 (0.85–0.99)	0.97 (0.85–0.99)
Specificity (95% CI)	0.83 (0.64–0.93)	0.80 (0.64–0.90)	0.80 (0.66–0.89)
**SP-A/serum, ng/mL**			
Cut-off value	16.10	73.60	48.00
Sensitivity (95% CI)	0.04 (0.01–0.19)	0.46 (0.29–0.65)	0.73 (0.54–0.86)
Specificity (95% CI)	0.86 (0.60–0.96)	0.81 (0.63–0.92)	0.81 (0.67–0.90)
**SP-D/plasma, ng/mL**			
Cut-off value	143.80	136.40	100.50
Sensitivity (95% CI)	0.88 (0.73–0.95)	0.88 (0.73–0.95)	0.91 (0.76–0.97)
Specificity (95% CI)	0.83 (0.64–0.93)	0.80 (0.64–0.90)	0.80 (0.66–0.89)
**CEA/plasma, ng/mL**			
Cut-off value	31.30	38.60	5.70
Sensitivity (95% CI)	0.42 (0.27–0.59)	0.36 (0.22–0.53)	0.13 (0.06–0.26)
Specificity (95% CI)	0.83 (0.64–0.93)	0.80 (0.64–0.90)	0.82 (0.66–0.91)
**CYFRA/plasma, ng/mL**			
Cut-off value	1.60	2.00	1.50
Sensitivity (95% CI)	0.76 (0.59–0.87)	0.73 (0.56–0.85)	0.76 (0.59–0.87)
Specificity (95% CI)	0.83 (0.64–0.93)	0.80 (0.64–0.90)	0.89 (0.77–0.95)
**LDH/serum, U/L**			
Cut-off value	178.00	189.00	193.00
Sensitivity (95% CI)	0.88 (0.73–0.95)	0.76 (0.59–0.87)	0.73 (0.56–0.85)
Specificity (95% CI)	0.83 (0.64–0.93)	0.80 (0.64–0.90)	0.80 (0.66–0.89)
**GM-CSF/plasma,pg/mL**			
Cut-off value	0.19	0.19	0.19
Sensitivity (95% CI)	1.00 (0.86–1.00)	1.00 (0.90–1.00)	1.00 (0.92–1.00)
Specificity (95% CI)	0.81 (0.65–0.91)	0.81 (0.65–0.91)	0.81 (0.65–0.91)
**GM-CSF autoantibodies/ plasma, ngeq/mL**			
Cut-off value	2223.00	133.00	2223.00
Sensitivity (95% CI)	1.00 (0.90–1.00)	1.00 (0.90–1.00)	1.00 (0.90–1.00)
Specificity (95% CI)	1.00 (0.86–1.00)	0.92 (0.78–0.97)	1.00 (0.92–1.00)
**CD11b stimulation index/whole blood**			
Cut-off value	0.24	0.24	0.24
Sensitivity (95% CI)	0.92 (0.74–0.98)	0.92 (0.78–0.97)	0.96 (0.85–0.99)
Specificity (95% CI)	0.82 (0.66–0.91)	0.82 (0.66–0.91)	0.82 (0.66–0.91)
**pSTAT5 stimulation index/whole blood**			
Cut-off value	0.15	0.17	0.08
Sensitivity (95% CI)	1.00 (0.86–1.00)	1.00 (0.90–1.00)	1.00 (0.92–1.00)
Specificity (95% CI)	0.82 (0.66–0.91)	0.82 (0.66–0.91)	0.82 (0.66–0.91)

*aPAP* autoimmune pulmonary alveolar proteinosis, *CD11b* cluster of differentiation molecule 11b, *CEA* carcinoembryonic antigen, *CI* confidence interval, *COPD* chronic obstructive pulmonary disease, *CYFRA* cytokeratin-fragment, *GM-CSF* granulocyte–macrophage colony-stimulating factor, *KL-6* Krebs von den Lungen 6, *LDH* lactate dehydrogenase, *pSTAT5* phosphorylated signal transducer and activator of transcription 5, *SD* standard deviation, *SP-A* surfactant protein-A, *SP-D* surfactant protein-D.

#### Induced sputum

There were no clear between-group differences in biomarkers (Table 2). Wide reference ranges, due to high inter-individual variability, did not allow for useful distinction between study populations using any of the measured biomarkers in induced sputum samples (Table 4).

**Table 4:** Diagnostic and predictive power for aPAP-induced sputum sample biomarkers (specificity ≥ 80%) [per-protocol biomarker analysis].

	aPAP vs healthy	aPAP vs COPD	aPAP vs asthma
**KL-6/sputum, U/mL**			
Cut-off value	248.90	314.40	248.90
Sensitivity (95% CI)	0.55 (0.38–0.70)	0.52 (0.35–0.67)	0.55 (0.38–0.70)
Specificity (95% CI)	0.83 (0.64–0.93)	0.80 (0.64–0.90)	0.95 (0.84–0.99)
**SP-A/sputum, ng/mL**			
Cut-off value	3.90	6.50	1.70
Sensitivity (95% CI)	0.58 (0.41–0.73)	0.36 (0.22–0.53)	0.76 (0.59–0.87)
Specificity (95% CI)	0.83 (0.64–0.93)	0.80 (0.64–0.90)	0.80 (0.66–0.90)
**SP-D/sputum, ng/mL**			
Cut-off value	3.18	12.06	1.88
Sensitivity (95% CI)	0.67 (0.50–0.80)	0.52 (0.35–0.67)	0.70 (0.53–0.83)
Specificity (95% CI)	0.83 (0.64–0.93)	0.80 (0.64–0.90)	0.81 (0.67–0.90)
**CEA/sputum, ng/mL**			
Cut-off value	63.30	55.50	47.30
Sensitivity (95% CI)	0.16 (0.07–0.32)	0.62 (0.45–0.76)	0.22 (0.11–0.39)
Specificity (95% CI)	0.83 (0.64–0.93)	0.81 (0.65–0.91)	0.80 (0.66–0.90)
**CYFRA/sputum, ng/mL**			
Cut-off value	Not calculable	11.70	Not calculable
Sensitivity (95% CI)		0.30 (0.17–0.47)	
Specificity (95% CI)		0.81 (0.65–0.91)	
**GM-CSF/sputum, pg/mL**			
Cut-off value	0.08	0.11	0.10
Sensitivity (95% CI)	0.82 (0.66–0.91)	0.76 (0.59–0.87)	0.79 (0.62–0.89)
Specificity (95% CI)	0.83 (0.61–0.94)	0.82 (0.66–0.91)	0.82 (0.67–0.91)

*aPAP* autoimmune pulmonary alveolar proteinosis, *CEA* carcinoembryonic antigen, *CI* confidence interval, *COPD* chronic obstructive pulmonary disease, *CYFRA* cytokeratin-fragment, *GM-CSF* granulocyte–macrophage colony-stimulating factor, *KL-6* Krebs von den Lungen 6, *SP-A* surfactant protein-A, *SP-D* surfactant protein-D.

#### Exhaled breath condensate

All biomarker levels in exhaled breath condensate samples at baseline were below lower quantification limits (data not shown).

### Multivariate modeling

#### Biomarkers alone

In most cases, serum/plasma GM-CSF values were sufficient to differentiate between aPAP and other groups (Additional File 1: Table S4). In induced sputum samples, a combination of SP-A, SP-D, and CYFRA was necessary to discriminate between aPAP and asthma, and a combination of SP-D, CYFRA, CEA and GM-CSF was necessary to discriminate between aPAP and COPD.

#### Biomarkers plus BMI, age and lung function parameters

In serum/plasma samples, clinical factors did not add value, with GM-CSF being an adequate discriminating factor between patients with aPAP and all other groups (Additional File 1: Table S4). In induced sputum samples, between three and six factors were required to discriminate between patients with aPAP and other populations.

## Discussion

This exploratory study determined levels and reference ranges of 10 biomarkers potentially associated with aPAP (Table 5) to monitor the disease development in pulmonary alveolar proteinosis, including in patients treated with anti-GM-CSF antibodies. The biomarkers used may differ depending on whether an individual is being assessed for spontaneous or iatrogenic disease. As anti-GM-CSF therapies have been proposed or investigated in COPD and asthma^19–21^, these patient groups were also investigated since biomarkers may also be useful in patients at risk of developing aPAP induced by anti-GM-CSF therapy.

**Table 5:** Summary of aPAP diagnostic ability for pre-specified biomarkers.

	Serum/plasma	Induced sputum	Diagnostic ability
KL-6	Approximately ten-fold higher in patients with aPAP than all other groups	Approximately ten-fold higher in patients with aPAP than all other groups	*Very good aPAP identifier in plasma samples* Less clear in induced sputum samples due to high inter-individual variability
SP-A	Similar in all four groups	Similar in all four groups	No clear difference between groups in serum or induced sputum
SP-D	Approximately three- to four-fold higher in patients with aPAP than all other groups	Approximately four- to 20-fold higher in patients with aPAP than all other groups	*Can be used as an aPAP identifier in plasma samples* Results not as good in induced sputum samples due to very high inter-individual variability
CEA	Slightly higher in patients with aPAP than all other groups	Approximately two-fold higher in patients with COPD than all other groups	No clear distinction between groups in plasma or induced sputum samples
CYFRA	Approximately five-fold higher in patients with aPAP than all other groups	Approximately two- to three-fold higher in patients with COPD than all other groups	*Can be used as an aPAP identifier in plasma samples* Not possible to distinguish aPAP using induced sputum samples
LDH	Increased by approximately 50% in patients with aPAP than all other groups	Not analyzed	Can be used as an aPAP identifier in serum samples
GM-CSF	Approximately five-fold lower in patients with aPAP than all other groups	Approximately 50- to 60-fold higher in patients with aPAP than all other groups	*Very good aPAP identifier in plasma samples* Utility in patients receiving anti-GM-CSF antibodies therapeutically not clear Lower sensitivity in induced sputum samples due to high inter-individual variability
GM-CSF autoantibodies	Very high in patients with aPAP but below limit of quantification in all other groups	Not analyzed	*Extremely good aPAP identifier in plasma samples* (in patients not receiving anti-GM-CSF antibodies)
GM-CSF-mediated CD11b expression	Approximately seven- to ten-fold lower in patients with aPAP than all other groups	Not analyzed	*Good aPAP identifier in whole blood samples* Utility in patients receiving anti-GM-CSF antibodies therapeutically not clear
GM-CSF-mediated pSTAT5 expression	Substantially lower in patients with aPAP than all other groups	Not analyzed	*Good aPAP identifier in whole blood samples* Utility in patients receiving anti-GM-CSF antibodies therapeutically not clear

*aPAP* autoimmune pulmonary alveolar proteinosis, *CD11b* cluster of differentiation molecule 11b, *CEA* carcinoembryonic antigen, *COPD* chronic obstructive pulmonary disease, *CYFRA* cytokeratin-fragment, *GM-CSF* granulocyte–macrophage colony-stimulating factor, *KL-6* Krebs von den Lungen 6, *LDH* lactate dehydrogenase, *pSTAT5* phosphorylated signal transducer and activator of transcription 5, *SP-A* surfactant protein-A, *SP-D* surfactant protein-D.

GM-CSF autoantibodies in plasma clearly differentiated patients with aPAP, healthy volunteers and patients with COPD or asthma without aPAP: patients with aPAP had high levels, as expected, while levels in other study populations were too low to quantify. However, it can be surmised that this biomarker will not be useful for patients receiving anti-GM-CSF antibodies therapeutically or patients with non-autoimmune pulmonary alveolar proteinosis whose condition is not characterized by GM-CSF autoantibodies^2^. GM-CSF, CD11b and pSTAT5 in plasma/serum were also good aPAP identifiers; these were all lower in patients with aPAP than in other study populations. Although GM-CSF levels in patients with aPAP were reduced in plasma samples, they were relatively elevated in sputum. One explanation may be that GM-CSF autoantibodies decrease the amount of free GM-CSF in plasma without affecting levels in sputum. KL-6 levels were increased in patients with aPAP, confirming previous findings^12,22–24^, but KL-6 is also elevated in other disease states^16,17^ and so may be less useful as an identifier of aPAP. The derived reference ranges for SP-A, SP-D, LDH, CEA and CYFRA either partially or completely overlapped across the different study populations; distinguishing aPAP based on just one of these biomarkers would be unreliable. None of the biomarkers analyzed in induced sputum was a suitable aPAP identifier in its own right, and biomarkers could not be detected in exhaled breath condensate.

Lung function findings from this study agree with published observations^4,14,22,25–27^. In patients with aPAP, DL_CO_ is frequently reduced, whereas FVC and FEV_1_ are generally within normal limits (although some patients with aPAP show decreased FVC)^26,28^. In patients with aPAP, DL_CO_% predicted correlates well with disease severity and FVC% predicted correlates to a lesser extent, whereas reductions in lung volume and airflow are minimal^4,29^. Results from the symptom questionnaires support the findings of the pulmonary function tests, with mean scores on the Asthma Control Questionnaire (ACQ), COPD Assessment Test (CAT) and St. George’s Respiratory Questionnaire (SGRQ) highest/worst for patients with asthma or COPD with severe disease or experiencing severe exacerbation.

This study was not aimed at evaluating changes in biomarkers as a function of disease progression as the interval to assess change over time was too short to expect any significant change in aPAP biomarkers. Results of multivariate modeling showed that a low level of GM-CSF in plasma alone was enough to differentiate between patients with aPAP and other study groups in most cases. When body mass index (BMI), age, and pulmonary function were considered, plasma GM-CSF level was sufficient to discriminate between aPAP and healthy volunteers, COPD or asthma in all cases. It is not clear if patients receiving anti-GM-CSF antibodies therapeutically would also have lower plasma GM-CSF or the discriminative value therein for aPAP. These findings are a first step to support the clinical development of anti-GM-CSF antibody therapies in patients with rheumatoid arthritis. Patients receiving anti-GM-CSF antibodies are theoretically at increased risk of aPAP^8^, and administration of GM-CSF-autoantibodies from a pulmonary alveolar proteinosis patient has been shown to induce pathologic manifestations of pulmonary alveolar proteinosis in healthy macaques^30^. Measuring plasma levels of GM-CSF, with or without other clinical variables, may help to identify patients most at risk of aPAP, or differentiate aPAP from worsening of existing condition. It will need to be determined whether patients receiving anti-GM-CSF antibodies have a reduction in plasma GM-CSF. If not, this could be the next step in utilization as a risk marker for aPAP in those patients.

This study has some limitations and findings are exploratory in nature. The patient populations had some differences in baseline characteristics. Due to the limited number of participants, biomarker reference ranges should be taken as a first estimate, and no confidence intervals were calculated for the limits of the reference ranges. More data will be needed to establish reliable biomarker reference ranges. Additionally, the risk assessment for aPAP is still unclear, as the time taken from development of low GM-CSF plasma levels to disease onset is unknown.

Accurate monitoring for aPAP is important in an era of anti-GM-CSF antibody therapies. In this study, plasma GM-CSF was a key marker (secondary to GM-CSF autoantibodies) for differentiating patients with aPAP from healthy volunteers and patients with COPD or asthma without aPAP. Other markers were less useful than GM-CSF for diagnostic purposes but may be interesting to research in combination as markers of potential aPAP development. Assessment of plasma GM-CSF may be sufficient to identify patients receiving anti-GM-CSF antibodies who are at increased risk of developing aPAP and require further medical evaluation. In this study, chest computed tomography findings along with anti-GM-CSF antibody positivity were used for confirmation of a diagnosis of aPAP, although these criteria alone may not be sufficient for definite diagnosis of aPAP^2^. Further study of plasma GM-CSF levels in patients receiving anti-GM-CSF antibodies may help define the relationship between plasma GM-CSF and aPAP risk and delineate the usefulness of such an approach.

## Methods

### Study design

This exploratory, prospective, observational study was conducted at a single Italian center over 120 days (between October 2013 and September 2015). Longitudinal evaluation of pulmonary function and biomarkers in different biofluids was performed in four groups: aPAP, healthy volunteers, patients with COPD, or patients with asthma. Anti-GM-CSF therapies have been investigated in asthma and COPD and could theoretically induce iatrogenic PAP. Patients with asthma and COPD not treated with GM-CSF were therefore included as a reference base.

The study was carried out in accordance with the principles of the Declaration of Helsinki, the International Conference on Harmonization for Good Clinical Practice, and local legal and regulatory requirements. All participants provided written informed consent prior to study participation. The local authority that approved the study on 11 February 2013 was the Ethics Committee at IRRCS Policlinico San Matteo Hospital Foundation (approval number: P-20120042069).

### Study participants

Patients with COPD were aged 40–70 years; all other participants were aged 18–70 years at study entry. All participants were required to have a BMI between 18 and 35 kg/m^2^. Definitions of disease severity for patients with aPAP, COPD and asthma are described in Additional file 1: Supplementary Appendix 1.

#### Patients with aPAP

Patients had a current diagnosis of aPAP with consistent computed tomography findings (crazy-paving pattern in the lungs) and elevated levels (≥ 5 μg/mL) of GM-CSF autoantibodies. aPAP was classified according to partial pressure of oxygen (PaO_2_) volumes as mild (asymptomatic; PaO_2_ ≥ 70 mmHg), moderate (PaO_2_ ≥ 60 and < 70 mmHg), or in stable remission (asymptomatic; PaO_2_ ≥ 70 mmHg with a whole lung lavage conducted 1–2 months prior to study entry). Patients with PaO_2_ < 60 were not included.

#### Healthy volunteers

Participants had normal values on pulmonary function tests at screening, were shown to be healthy by clinical laboratory tests and vital signs, had not received GM-CSF in the previous 4 weeks, and could not have a diagnosis of aPAP.

#### Patients with COPD

Patients had a current diagnosis of COPD of at least 3 months’ duration. COPD was diagnosed in accordance with Global Initiative for Chronic Obstructive Lung Disease guidelines and was classified as mild, moderate or severe^31^. Patients were either stable for at least 1 month, or experiencing a severe exacerbation (defined as symptoms, starting within 5 days prior to screening, requiring hospitalization and/or change in COPD treatment for at least 3 days). Patients were required to be current smokers or ex-smokers with a smoking history of > 10 pack-years, and not to have received GM-CSF in the previous 4 weeks and not to have a diagnosis of aPAP.

#### Patients with asthma

Patients had a current diagnosis of asthma of at least 3 months’ duration and were diagnosed before 40 years of age. Asthma was diagnosed in accordance with Global Initiative for Asthma guidelines, and classified as mild, moderate or severe^32^. Patients were either stable for at least 1 month, or experiencing a severe exacerbation (defined as symptoms, starting within 5 days prior to screening, requiring actual or intended treatment with systemic corticosteroids or at least a doubling of previous daily doses of systemic corticosteroids, for at least 3 days). Patients were non-smokers or ex-smokers with a smoking history of ≤ 10 pack-years who had stopped smoking at least 1 year prior to enrollment, had not received GM-CSF in the previous 4 weeks and could not have a diagnosis of aPAP.

### Clinical assessments

#### Symptom-based questionnaires

Self-administered tests, designed to measure patient perception of health impairment, were completed by patients with COPD or asthma at baseline, Day 60 and Day 120, prior to pulmonary function tests and any other study procedures. Patients with aPAP and healthy volunteers did not complete symptom-based questionnaires. Patients with COPD completed the CAT^33^ followed by the SGRQ^34^. Patients with asthma completed the ACQ^35^.

#### Pulmonary function tests

DL_CO_ was measured in all participants at baseline, Day 60 and Day 120. Values are reported for DL_CO_% predicted and absolute measured values corrected for hemoglobin. Spirometry (FEV_1_ and FVC) was performed in all participants at baseline, Day 60 and Day 120. Absolute FEV_1_, FEV_1_% predicted and FVC% predicted values are also reported.

### Biomarker assessments

Ten pre-specified biomarkers were analyzed: KL-6, SP-A and SP-D by quantitative sandwich enzyme immunoassay, LDH by standardized colorimetric assay, CEA and CYFRA by immunoassay, GM-CSF and GM-CSF autoantibodies by enzyme-linked immunosorbent assay, and CD11b and pSTAT5 by fluorocytometry (Additional file 1: Supplementary Appendix 2 and Table S1).

Biomarker levels were measured in biofluid samples (serum/plasma, induced sputum and exhaled breath condensate) taken at baseline, Day 60 and Day 120 (Additional file 1: Supplementary Appendix 3 and Table S1). Induced sputum was collected from participants with an FEV_1_% predicted ≥ 50%. Stimulated and unstimulated values of GM-CSF-mediated CD11b and pSTAT5 expression on neutrophilic granulocytes were measured in whole blood, and stimulation index was calculated as (stimulated value – unstimulated value)/unstimulated value.

Using the measured biomarker data in blood and induced sputum, reference ranges were calculated for each of the biomarkers in each of the four trial populations (Additional file 1: Supplementary Appendix 4).

### Safety outcomes

The safety of study procedures was assessed based on vital signs, physical examination, clinical laboratory tests and incidence of adverse events.

### Statistical methods

#### Primary analyses

Descriptive statistics were calculated for clinical outcomes and biomarker values. Reference intervals were determined for each biomarker using methods described in the Clinical and Laboratory Standards Institute guideline^36^.

To analyze the potential diagnostic ability of biomarkers to distinguish between participants in the four study populations, pairwise comparisons were made for biomarker values in blood and induced sputum samples where applicable. Cut-off values were determined to estimate sensitivity to detect aPAP for a given specificity (≥ 80%). Receiver-operating characteristic curves were plotted to quantify the ability of each biomarker to predict the presence or absence of aPAP.

#### Secondary analyses

Multivariate statistical prediction models based on logistic regression were developed using: (a) biomarker values alone, and (b) biomarker values in conjunction with established clinical variables (BMI, age, lung function parameters) as potential predictors. All biomarkers apart from GM-CSF autoantibody level were included in the models, as GM-CSF autoantibody concentrations were higher in patients with aPAP than in all other study populations. Inclusion would have added too much bias since it would have been the only predictor in the model resulting in perfect separation. The aim was to identify alternatives to GM-CSF autoantibodies, as these were also part of the diagnostic inclusion criteria for patients with aPAP.

### Ethics approval and consent to participate

The study was carried out in accordance with the principles of the Declaration of Helsinki, the International Conference on Harmonization for Good Clinical Practice, and local legal and regulatory requirements. All participants provided written informed consent prior to study participation. The local authority that approved the study on 11 February 2013 was the Ethics Committee at IRRCS Policlinico San Matteo Hospital Foundation (approval number: P-20120042069).

## Supplementary Information

Supplementary Information.

## Data Availability

Generally, Boehringer Ingelheim makes data that support the findings of studies available from https://trials.boehringer-ingelheim.com/ but restrictions apply to the availability of these data where anonymization cannot be ensured. This is particularly the case for single-center studies like the study discussed in this publication. Therefore, in this case, Boehringer Ingelheim cannot share any additional data.

## Abbreviations

ACQ: Asthma Control Questionnaire
aPAP: Autoimmune pulmonary alveolar proteinosis
BMI: Body mass index
CAT: COPD Assessment Test
CD11b: Cluster of differentiation molecule 11b
CEA: Carcinoembryonic antigen
COPD: Chronic obstructive pulmonary disease
CYFRA: Cytokeratin fragment
DL_CO_: Diffusing capacity of the lungs for carbon monoxide
FEV_1_: Forced expiratory volume in 1 s
FVC: Forced vital capacity
GM-CSF: Granulocyte-macrophage colony-stimulating factor
KL-6: Krebs von den Lungen 6
LDH: Lactate dehydrogenase
PaO_2_: Partial pressure of oxygen
pSTAT5: Phosphorylated signal transducer and activator of transcription 5
SGRQ: St. George’s Respiratory Questionnaire
SP-A: Surfactant protein-A
SP-D: Surfactant protein-D
