# Supplementary material for: An exploratory study investigating biomarkers associated with autoimmune pulmonary alveolar proteinosis (aPAP)
Source: Sci Rep. 2022 May 24;12:8708. doi: 10.1038/s41598-022-11446-8 (PMC9130212; doi:10.1038/s41598-022-11446-8)
Supplement: Supplementary file 1 — Supplementary Information. [file 41598_2022_11446_MOESM1_ESM.docx]

**Supplementary information**

**An exploratory study investigating biomarkers associated with autoimmune pulmonary alveolar proteinosis (aPAP)**

Ilaria Campo (PhD), Federica Meloni (PhD), Martina Gahlemann (MD), Wiebke Sauter (PhD), Carina Ittrich (PhD), Corinna Schoelch (PhD), Bruce C. Trapnell (MD), Abhya Gupta (PhD)

**Appendix 1 – Definitions of disease severity**

*aPAP*

Mild: no symptoms, PaO_2_ ≥70 mmHg.

Moderate: PaO_2_ ≥60 and <70 mmHg.

*COPD*

Mild: post-bronchodilator FEV_1_ predicted ≥80% and FEV_1_/FVC <70%.

Moderate: 50% ≤post-bronchodilator FEV_1_ predicted <80% and FEV_1_/FVC <70%, no chronic respiratory failure.

Severe: 30% ≤post-bronchodilator FEV_1_ predicted <50% and FEV_1_/FVC <70%, no chronic respiratory failure.

Severe COPD exacerbation: start of symptoms not earlier than 5 days prior to Visit 1 and requiring a hospitalization and/or change in COPD treatment (antibiotics, and/or systemic steroids), actual or intended, with a duration of 3 days or more.

*Asthma*

Mild: GINA treatment step 1 or 2 and ACQ <0.75.

Moderate: GINA treatment step 3 and ACQ >1.5, with pre-bronchodilator FEV_1_ predicted ≥50%.

Severe: GINA treatment step 4 or 5 and ACQ >1.5, with pre-bronchodilator FEV_1_ predicted ≥50%. Severe asthma exacerbation: start of symptoms not earlier than 5 days prior to Visit 1 and actual or intended treatment with systemic corticosteroids for at least 3 days.

**Appendix 2 – Sites of biomarker analyses**

Fluorescence-activated cell sorting (FACS) assays for the analyses of CD11b and pSTAT5 were completed at the trial site (Institute for Respiratory Disease, University of Pavia, Fondazione IRCCS Policlinico San Matteo, Pavia, Italy). Other biomarker analyses were performed at one of two external contract research organizations (Icon Development Solutions, Whitesboro, TX, USA, and Quest Diagnostic Nichols Institute, Valencia, CA, USA) or at Boehringer Ingelheim International GmbH & Co. KG, Biberach, Germany.

**Appendix 3 – Obtainment of biofluids**

*Serum/plasma*

Samples were obtained at baseline, Day 60 and Day 120 of the trial. At each visit, approximately 50 mL blood was taken from a forearm vein of all participants.

*Induced sputum*

Samples were obtained at baseline, Day 60 and Day 120. In patients who were not at risk of bronchospasm (>95% of patients), general sputum induction was used to obtain samples. This method was not used in patients who were at risk of bronchospasm (FEV_1_ value less than 50% of the predicted normal value).

*Exhaled breath condensate*

Samples were obtained at baseline, Day 60 and Day 120 after a resting time of at least 10 minutes and before any pulmonary function test measurements in order to avoid traces of salbutamol. Participants were to refrain from eating or drinking (other than water) for at least 3 hours before collection. Collection time was 15 minutes and the collection volume was approximately 2 mL.

**Appendix 4 – Calculation of biomarker reference ranges in serum/plasma or whole blood samples, at baseline (per-protocol biomarker analysis sets)**

Ranges were calculated for each of the biomarkers for each of the four trial populations, unless more than 20% of the data points were below or above the limit of quantification.

As more than 20% of the data points were below or above the limit of quantification, it was not possible to calculate accurate reference ranges for the CYFRA and GM-CSF autoantibodies data.

**Table S1.** **Pre-specified biomarker assessments**

| **Analyte** | **Biofluid** | **Assay** |
| --- | --- | --- |
| KL-6 | Plasma  Induced sputum  Exhaled breath condensate | Quantitative sandwich enzyme immunoassay |
| SP-A | Serum  Induced sputum  Exhaled breath condensate | Quantitative sandwich enzyme immunoassay |
| SP-D | Plasma  Induced sputum  Exhaled breath condensate | Quantitative sandwich enzyme immunoassay |
| DPPC* | Induced sputum  Exhaled breath condensate | LC/MS-MS assay |
| CEA | Plasma  Induced sputum  Exhaled breath condensate | Immunoassay |
| CYFRA | Plasma  Induced sputum  Exhaled breath condensate | Immunoassay |
| LDH | Serum | Standardized colorimetric assay |
| GM-CSF | Plasma  Induced sputum  Exhaled breath condensate | Fluorescence immunoassay (Erenna) |
| GM-CSF autoantibodies | Plasma | ELISA |
| GM-CSF-mediated CD11b expression | Whole blood | FACS |
| GM-CSF-mediated pSTAT5 expression | Whole blood | FACS |

*No data are available for DPPC, as DPPC was absorbed by any plastic material used in the assay set-up and therefore could not be measured in any of the matrices.

CD11b, cluster of differentiation molecule 11b; CEA, carcinoembryonic antigen; CYFRA, cytokeratin-fragment; DPPC, dipalmitoylphosphatidylcholin; ELISA, enzyme-linked immunosorbent assay; FACS, fluorescence-activated cell sorting; GM-CSF, granulocyte-macrophage colony-stimulating factor; KL-6, Krebs von den Lungen 6; LC/MS-MS, liquid chromatography with tandem mass spectrometry; LDH, lactate dehydrogenase; pSTAT5, phosphorylated signal transducer and activator of transcription 5; SP-A, surfactant protein-A; SP-D, surfactant protein-D.

**Table S2.** **Pulmonary function parameters at baseline, Day 60 and Day 120 (per-protocol biomarker analysis sets)**

|  | **aPAP  (*n* = 28–33)** | **Healthy  (*n* = 20–24)** | **COPD  (*n* = 32–35)** | **Asthma  (*n* = 36–44)** |
| --- | --- | --- | --- | --- |
| ***DL_CO_% predicted, mean (SD) %*** | | | | |
| Baseline  Day 60  Day 120 | 64.2 (17.1)  66.1 (19.7)  64.9 (18.4) | 88.3 (14.6)  88.3 (15.9)  83.8 (15.1) | 67.3 (20.0)  67.0 (21.6)  65.6 (20.4) | 91.8 (15.0)  87.3 (11.7)  87.2 (13.6) |
| ***DL_CO_ corrected for Hb, mean (SD) mmol/min/kPa*** | | | | |
| Baseline  Day 60  Day 120 | 3.8 (1.1)  4.0 (1.3)  3.8 (1.3) | 5.7 (1.4)  5.8 (1.5)  5.5 (1.5) | 3.5 (1.1)  3.5 (1.2)  3.4 (1.1) | 5.4 (1.6)  5.1 (1.4)  5.1 (1.5) |
| ***FEV_1_, mean (SD) L*** | | | | |
| Baseline  Day 60  Day 120 | 2.92 (0.81) 2.88 (0.84) 2.87 (0.83) | 3.86 (0.86) 3.82 (0.89) 3.80 (0.88) | 1.65 (0.61) 1.61 (0.58) 1.58 (0.58) | 2.89 (0.80) 2.87 (0.86) 2.83 (0.85) |
| ***FEV_1_% predicted, mean (SD) %*** | | | | |
| Baseline  Day 60  Day 120 | 90.1 (19.7)  87.9 (20.7)  88.9 (20.0) | 106.1 (8.6)  104.8 (8.4)  104.4 (8.8) | 60.7 (21.3)  59.1 (21.0)  58.0 (19.8) | 91.4 (15.3)  91.9 (14.4)  90.4 (14.5) |
| ***FVC% predicted, mean (SD) %*** | | | | |
| Baseline  Day 60  Day 120 | 93.6 (20.8)  91.7 (22.3)  92.8 (21.3) | 110.0 (11.2)  109.4 (10.8)  108.1 (11.9) | 84.2 (16.2)  80.0 (16.6)  78.5 (16.9) | 106.1 (14.1)  105.7 (13.5)  103.0 (13.7) |

Numbers of patients assessed differ slightly at each time point.

aPAP, autoimmune pulmonary alveolar proteinosis; COPD, chronic obstructive pulmonary disease; DL_CO_, diffusing capacity of the lungs for carbon monoxide; FEV_1_, forced expiratory volume in 1 second; FVC, forced vital capacity; Hb, hemoglobin; SD, standard deviation.

**Table S3. Potential correlations between lung parameters and biomarkers (per-protocol biomarker analysis sets)**

|  | **Healthy**  ***n* = 24** | **COPD**  ***n* = 36** | **Asthma**  ***n* = 45** | **aPAP**  ***n* = 33** |
| --- | --- | --- | --- | --- |
|  |  |  |  | **Spearman correlation coefficient (95%CI)** |
| **Blood biomarkers** |  |  |  |  |
| KL-6/DL_CO_^1^ | No correlation | No correlation | No correlation | −0.554  (−0.761, −0.226) |
| SP-A/DL_CO_^1^ | No correlation | No correlation | No correlation | −0.645  (−0.825, −0.324) |
| SP-D/DL_CO_^1^ | No correlation | No correlation | No correlation | −0.502  (−0.729, −0.157) |
| LDH/DL_CO_^1^ | No correlation | No correlation | No correlation | −0.661  (−0.824, −0.379) |
| KL-6/FVC%pred | No correlation | No correlation | No correlation | −0.558  (−0.753, −0.258) |

A correlation was assumed possible if the Spearman correlation coefficient was ≥0.5 or ≤-0.5 and the 95% CI did not include 0.

No possible correlations were seen, in any analysis set, between lung parameters and other blood biomarkers (CEA, CYFRA, GM-CSF free, GM-CSF autoantibodies, GM-CSF-mediated CD11b, GM-CS-mediated pSTAT5) or between lung parameters and any induced sputum biomarkers (KL-6, SP-A, SP-D, CEA, CYFRA, GM-CSF).

^1^DLco was corrected for hemoglobin.

aPAP, autoimmune pulmonary alveolar proteinosis; CEA, carcinoembryonic antigen; CI, confidence interval; CD11b, cluster of differentiation molecule 11b; COPD, chronic obstructive pulmonary disease; CYFRA, cytokeratin-fragment; DL_CO_, diffusing capacity of the lungs for carbon monoxide; FVC%pred, forced vital capacity percent predicted; GM-CSF, granulocyte macrophage colony-stimulating factor; KL-6, Krebs von den Lungen 6; LDH, lactate dehydrogenase; pSTAT5, phosphorylated Signal Transducer and Activator of Transcription 5; SP-A, surfactant protein-A; SP-D, surfactant protein-D.

**Table S4: Diagnostic and predictive power of multivariate prediction models for comparison of aPAP patients versus the other trial populations (specificity of ≥80%) [per-protocol biomarker analysis]**

| **Sets of potential discriminators** | **aPAP versus healthy** | **aPAP versus asthma** | **aPAP versus COPD** |
| --- | --- | --- | --- |
| **Biomarkers in blood and induced sputum**  Discriminating factors  Sensitivity (%)^1^  Specificity (%)^1^  Correct classification (%)^1^ | GM-CSF (b)  93.8  87.5  91.1 | GM-CSF (b)  96.9  93.0  94.7 | GM-CSF (b)  96.9  82.9  89.6 |
| **Biomarkers in blood**  Discriminating factors  Sensitivity (%)^1^  Specificity (%)^1^  Correct classification (%)^1^ | GM-CSF (b)  93.8  87.5  91.1 | GM-CSF (b)  96.9  93.0  94.7 | GM-CSF (b)  96.9  82.9  89.6 |
| **Biomarker in induced sputum**  Discriminating factors  Sensitivity (%)^1^  Specificity (%)^1^  Correct classification (%)^1^ | GM-CSF (is)  81.8  83.3  82.4 | SP-A (is), SP-D (is),  CYFRA (is)  78.1  90.2  84.9 | SP-D (is), CYFRA (is),  CEA (is), GM-CSF (is)  81.3  87.1  84.1 |
| **Biomarkers in blood, induced sputum and clinical variables^2^**  Discriminating factors  Sensitivity (%)^1^  Specificity (%)^1^  Correct classification (%)^1^ | GM-CSF (b)  93.8  87.5  91.1 | GM-CSF (b)  96.9  93.0  94.7 | GM-CSF (b)  96.9  82.9  89.6 |
| **Biomarkers in blood and clinical variables^2^**  Discriminating factors  Sensitivity (%)^1^  Specificity (%)^1^  Correct classification (%)^1^ | GM-CSF (b)  93.8  87.5  91.1 | GM-CSF (b)  96.9  93.0  94.7 | GM-CSF (b)  96.9  82.9  89.6 |
| **Biomarker in induced sputum and clinical variables^2^**  Discriminating factors  Sensitivity (%)^1^  Specificity (%)^1^  Correct classification (%)^1^ | DL_CO_, CEA (is),  GM-CSF (is), age  75.0  87.5  79.5 | DL_CO_, KL-6 (is), SP-D (is), CEA (is), CYFRA (is), age  89.3  85.3  87.1 | FEV_1_%, FVC%, GM-CSF (is)  100.0  87.5  93.8 |

^1^For the respective probability cut-off values needed to achieve a specificity of ≥80%.

^2^BMI, age, and the lung function parameters DL_CO_ corrected for hemoglobin, FEV_1_% predicted, and FVC% predicted.

b, blood sample (serum, plasma or whole blood); BMI, body mass index; CEA, carcinoembryonic antigen; COPD, chronic obstructive pulmonary disease; CYFRA, cytokeratin fragment; DL_CO_, diffusing capacity of the lungs for carbon monoxide; FEV_1_, forced expiratory volume in 1 second; FVC, forced vital capacity; GM-CSF, granulocyte-macrophage colony-stimulating factor; is, induced sputum sample; KL-6, Krebs von den Lungen-6; aPAP, autoimmune pulmonary alveolar proteinosis; SP-A, surfactant protein-A; SP-D, surfactant protein-D.
